# "The Hospital" Nursing Mirror

**Published:** 1898-11-26

**Authors:** 


### "The Hospital" Nursing Mirror.

Being the Nursing Section of "The Hospital."

[Contributions for this Section of "The Hospital" should be addressed to the Editor, The Hospital, 28 & 29, Southampton Street, Strand, London, W.C., and should have the word "Nursing" plainly written in left-hand top corner of the envelope.]

### News from the Nursing World.

### THE ALEXANDRA NURSES.

The nurses employed by the Soldiers and Sailors' Families' Association will henceforth, by the permission of H.R.H. the Princess of Wales, be known as the "Alexandra Nurses." The Princess is the president of this association, which was founded in 1885, and which has several branches for the benefit of the families of both officers and men employed in the Army and Navy. The nursing branch was inaugurated in 1892, and supplies qualified district nurses to attend the families of soldiers and sailors in large garrison and seaport towns. The Princess's approval, therefore, of the new title is but a fresh instance of her continual interest, not only in the objects for which the society was founded, but in all that appertains to the work of nursing and those engaged in it.

### FOR SERVICE RENDERED.

The reward of merit is sweet, and we heartily congratulate the three nurses who have had the privilege of nursing the sick during the recent Soudan campaign upon the recognition of their services by her Majesty. The three ladies who have received the decoration of the Royal Red Cross are Miss Sarah Emily Webb, who is an army sister, and still on duty in the hospital at Alexandria ; Miss Amy Florence Grist, who is also an army sister, and stationed at Cairo ; and Miss Elizabeth Geddes, who, with two others, was sent out under the auspices of the National Aid Society for the Sick and Wounded in War. Miss Geddes, and the two nurses who went out with her, are members of the Nurses' Co-operation, 8, Old Cavendish Street, W. She is the daughter of a Fife schoolmaster, and began her training ten years ago in the Dundee Royal Infirmary, where she gained first-class certificates. The Red Cross ship, the "Mayflower," made three trips up the Nile, and brought down those of the sick and wounded who were unfit for the 700 miles of railway journey. It was of these that the Red Cross nurses had charge. Miss Geddes prolonged her stay in Egypt, acting as night superintendent at the Citadel Hospital, Cairo.

### AN ITINERANT SUPERINTENDENT.

Dr. Browneigg, Bishop of Ossory, Ireland, has lately made a novel proposition to the Guardians of the Castlecomer Union, Kilkenny. So far it has met with unanimous support. His lordship suggests that a lady, who is a fully-trained competent nurse with six years' experience in the London hospitals, shall visit for a time the workhouse infirmaries and other similar public institutions, and instruct the nurses in the latest nursing methods in vogue. No implication of defective nursing is implied ; the itinerant superintendent—for such this visiting nurse will practically be—merely places within the grasp of others less favoured in opportunity than herself her knowledge and experience of the latest progress in the art of nursing. The Bishop purposes to pay her salary out of his own pocket, the Guardians becoming responsible for residence and maintenance only. The success or failure of this plan rests of necessity upon the personal calibre of the itinerant superintendent. A woman with tactful, sympathetic nature would be welcomed (especially with the Bishop's introduction) with all the warm-hearted hospitality for which the Irish are famous ; but it is more than doubtful whether even such an introduction could secure cordial relations if there were a suspicion of patronage or interference in the visitor's bearing.

### SUNDERLAND INFIRMARY NURSES' HOME.

On Saturday last a new Nurses' Home in connection with the Sunderland Infirmary was opened by Alderman Stanfield Richardson, in the unavoidable absence of Mrs. Richardson through sickness. Half the sum collected in Sunderland in commemoration of the Diamond Jubilee, amounting to £2,381, was awarded to the infirmary, and has been spent by the authorities in the building of this new home. The architects are Messrs. Potts, and the contractors Messrs. Walter Scott and Son.

### NURSING ORGANISATION IN CAPETOWN.

According to the account given at the first annual meeting of the Victoria Nurses' Institute, nursing in Capetown was, till a short year ago, characterised by an utter lack of organisation. To seek a trained nurse was somewhat like hunting a needle in a bottle of hay. A few chemists kept a list of nurses who did not think it worth while to notify any change of address, or whether they had work or not. But all this has been changed, and changed by the work of women. Miss Martha Miller, a certificated nurse, in conjunction with an energetic committee, of which Mrs. J. Rose Innis is the president, established first a register of all nurses whose certificates entitled them to be examined by the Colonial Medical Council. The next step was the purchase and fitting up of a house in Hof Street by public subscription as their permanent head-quarters and residence. Thus was inaugurated the Victoria Nurses' Institute as a Diamond Jubilee memorial. The work was begun without any nurses for the poor, but nurses cannot fail to hear the cry of sickness for help and not respond. Mrs. Rawbone and Miss Kearns organised District No. 1, and collected £60 a year in a hundred small subscriptions to pay the salary of a district nurse. The institution provided residence and maintenance. Other claims were put forward, and a second nurse was appointed, with the result that the terrible need of more nurses became apparent, especially for maternity work. Consequently, when the institute presented its first report to the public it was to ask in no uncertain voice for funds to provide nurses, a maternity hospital, and to enlarge the home.

We feel very certain, after reading the report, that that money will be forthcoming, and that the generosity of the matron, who has added a further term of six months' gratuitous service to the year's work she has already given, is but an ensample of the spirit that animates committee, nurses, and the public.

### ENGLISH GIRLS IN PARIS.

Miss Rhoda Mouncey, 45, Eastbourne Terrace, Hyde Park, the present secretary of the British and American Home in Paris, situated at 77, Avenue Wagram, originally founded for all English-speaking girls by Miss Ada Leigh, is asking for pecuniary help, as the work is unendowed, and many charitable agencies have sprung up around it. We confess that we mention it more with a view of giving our English nurses abroad, or on the point of going abroad, the address where they may obtain safe lodging and advice, than for the benefit of the undertaking, but fortunately in this case it seems possible to kill two birds with the same stone, and serve both nurses and charity at the same time.

### TO HELP IRISH LADIES.

In the press of new requirements, new calls on the public purse, one is apt to forget the causes that in a bygone, but not distant day, won the ready response of the warm-hearted. The sufferings of ladies because of the evil times through which Ireland has been passing are not less real now than they were then, and there are still 80 ladies on the pension list of the Irish Distressed Ladies' Fund. Eighty ladies, one 95 years old, and the income of the pension fund only £1,120! We sincerely trust that the present appeal set forth by the chairmen of the London and Irish Executive Committees will be warmly supported, and that enough money to continue the small sums allowed these ladies may thus be assured during the coming year.

### ST. LAURENCE, BIRMINGHAM.

The Vicar of St. Laurence, Birmingham, is endeavouring to form a Nurses' Home in his parish. It is one of the poorest in the town, the inhabitants earning a scanty livelihood as rag-pickers and kindred occupations. The death-rate is 40 in the thousand, and the population borders upon 8,000 persons. The greatness of the need has justified the Vicar in securing the services of two trained district nurses, and we hope that his efforts to secure the co-operation of his wealthier neighbours and friends will be successful, and that the salaries of the nurses will speedily be forthcoming. Contributions should be sent to the Vicar, St. Laurence Lodge, Dartmouth Street, Birmingham.

### CHARITY AT SWANSEA.

Half the proceeds of a charity performance of the "Circus Girl" at the Swansea Theatre has been devoted to the hospital of that town. The Mayor and Mayoress have worked hard to secure for it popular support, and a full audience was the result. The share falling to the hospital amounted to £50, to which must be added the sum realised by the sale of programmes.

### OUR CLOTHING DISTRIBUTION.

Another delightful parcel has just arrived for our Christmas Distribution of Clothing. It contains half-a-dozen children's flannel night dresses, four petticoats, two knitted bonnets, and a crossover; and the donor, Miss N. Tallack, writes: "I am greatly interested in the work, which, with a little assistance from others, has been a great pleasure." Our pleasure in receiving it is likewise great.

### THE NOVELIST NURSE.

The servants' hall is in possession of most of the family secrets, for be Jeames ever so log-like and his employers ever so discreet, somehow or other things that were supposed not to be known leak out to the annoyance of those whom they may concern. Bad as this is, it is nothing to the novelist nurse, who, according to a contemporary, sits over her unconscious patient note-book in hand, and jots down in shorthand the revelations of his delirium which she as promptly turns into copy and disposes of for cash. Such a nurse as is there described is, let us hope, merely the product of a morbid imagination, as but few nurses can write shorthand, and not very many can write for publication. The lax principle, however, which is at the root of such a breach of confidence is not so rare as one would wish. To turn into filthy lucre the knowledge which has been gained in the sick room would of course be recognised by every nurse as an outrage; but to turn it into gossip, and to wile away the tedious hours with tales about the cases one has nursed, is, we fear, a not altogether uncommon failing even with nurses who would scorn to turn such gossip into cash. The very suggestion, however, of the latter possibility raises the question, What is to be done to safeguard a delirious sick one against the unscrupulous nurse? In the first place the public should be more careful that the nurses it employs are of honourable character and high principle, qualities to be rated higher even than much training; secondly, the relatives and friends ought not to resign all the duties of the sick room to strangers. Nurses may find anxious relations a bore, and it may be their duty to prevent their causing any excitement in their patient's room, but when it is possible, it is but just and kind that they should be permitted to share in the ministering to their sick.

### SHORT ITEMS.

The foundation-stone of a Nurses' Home and of the "Peace Cottages"—the memorial at Hunton of Her Majesty's Diamond Jubilee—was laid a short time since by Mr. Thomas Duncombe Eden. The site is the gift of Mrs. Alexander, who has, in addition, provided for the laying on of hot water and other comforts for the future residents; whilst Mr. Eden has undertaken the building. The four cottages are intended as homes for old parishioners.—Sister Sykes, Lady Superintendent of the Barry Nursing Association, has resigned. The staff of nurses will remove to the Victoria Jubilee Nurses' Home, now in process of completion, about Christmas time. The home is off Court Road, Barry Docks.—£23, the result of the annual demonstration of the friendly, temperance, and trade societies of North London, was recently given to the treasurer of the North London Nursing Association at a public meeting called for the purpose.—How fast the times move! Ten years ago how few district nurses pursued their beneficent labours amongst the poor, yet already a correspondent of the local press speaks of the appointment of a nurse for Bulwell (Nottingham) as the removal of a "reproach"!

### Nursing in Diseases of the Throat, Nose, and Ear.

By MACLEOD YEARSLEY, F.R.C.S.Eng., Assistant Surgeon to the Royal Ear Hospital, Surgeon-in-Charge of the Department for Diseases of the Throat, Nose, and Ear, the Farringdon General Dispensary, Hon. Surgeon for Diseases of the Throat and Ear, the Governesses' Home, &c.

### VII.—THE AFTER TREATMENT OF NASAL OPERATIONS.

GENERALLY speaking, the after treatment of operations upon the nose and naso-pharynx does not materially differ from that of those upon other regions. There are, however, one or two minor points, attention to which will be of value both in regard to the success of the treatment and the comfort of the patient, and a few words thereon will not be amiss.

In our third article operations upon the nose and naso-pharynx were divided into three groups, and it will be as well if that classification be adhered to in speaking of after treatment.

### 1.—OPERATIONS ON THE NASAL CAVITIES.

Cautery operations may be dismissed at once; their after treatment does not concern the nurse, and can be carried out by the patient at home. Some surgeons leave such cases entirely alone, others prefer to have the nose gently syringed in the manner already described in Article III. When this is done care must be taken if a rubber splint has been inserted in the nostril not to disturb it, but to syringe gently along its side.

TURBINECTOMIES.—After operations for the removal of the whole or parts of the turbinal bones the surgeon will probably indicate the treatment he wishes carried out. When a complete turbinectomy has been done the bleeding is often very profuse, but can usually be controlled by plugging with strips of gauze when the operation is completed. These should not as a rule be disturbed until the patient is seen again by the surgeon. Turbinectomy has been followed by secondary hæmorrhage. Should this occur the nurse should at once send for the surgeon and, whilst waiting for his response, should endeavour to control the bleeding by plugging with gauze as indicated. There is said also to be some danger of septic trouble after complete turbinectomy, but this complication is, I think, a rare one; an opinion which, I believe, is shared by other rhinologists. Provided due precautions are taken at the operation to prevent sepsis such a complication should not occur, and both that and secondary hæmorrhage can be avoided by interfering as little as possible with the nasal cavities after an operation, and so ensuring to them the rest so essential to healing and guarding against the possible introduction of septic material. After removal of the posterior ends of the turbinals syringing should not be practised, for reasons that will be referred to later.

OPERATIONS ON THE SEPTUM.—Much of what has just been said regarding turbinectomy applies here; probably inaction is of more value than interference. Should the surgeon order syringing, it must be done along the sides of the splints, which must on no account be disturbed. After all severe nasal operations the patient generally experiences discomfort from the swelling of the nose and eyelids and excessive tear secretion. This passes off rapidly, and can be much alleviated by bathing and sponging with hot water.

As regards the removal of tumours, again there is little to be said. Hæmorrhage after the removal of polypi, &c., can be readily controlled by insufflation of tanno-gallic acid, the application of ice, or peroxide of hydrogen, and scarcely ever calls for plugging. When the growth removed has been of a malignant nature, its extirpation will have probably been carried out by means of one of the more serious resection operations, the after treatment of which will be at the direction of the surgeon, and belongs more to the province of general surgical nursing.

### II.—OPERATIONS UPON THE ACCESSORY SINUSES.

After operations upon the maxillary sinus, the nurse may be called upon to syringe daily through the opening made. The operations upon this sinus differ somewhat with different surgeons, but the method employed by my colleagues and myself at the Royal Ear Hospital may be taken as fairly typical of the methods employed. The cavity is opened either through the alveolus or through the canine fossa, and a spiral silver drainage tube inserted. The cavity is then washed out daily with antiseptic lotion (carbolic 1 in 40, boric acid, or permanganate of potassium). It is this washing out that concerns the nurse, and the proper method of doing it is as follows:—

The patient is seated, a towel is placed round the neck, and he holds a bowl beneath his chin. The drainage tube is removed from the alveolus and placed in 1 in 20 carbolic lotion. An indiarubber syringe, with a special nozzle of silver made the size of the drainage tube, is filled with the solution to be used (suitably warmed), and the nozzle inserted into the opening. The cavity is then gently washed out until the fluid which issues from the patient's nose is no longer discoloured or turbid. The drainage tube is then cleaned with a brush and reinserted.

The after treatment of empyema of the frontal sinus differs according to whether the surgeon employs a tube or not. In the latter case the after syringing will be done by the surgeon himself, and should not be carried out by a nurse. If, however, a drainage tube has been left in the passage from the sinus to the nose, the nurse may be required to irrigate the cavity through the tube once or twice daily with a weak antiseptic lotion (*e.g.*, boric acid gr. xx. to ℥i). This should be carried out in much the same way as that described for the maxillary sinus, except that the drainage tube must not be removed. The lotion must be suitably warmed before it is used, and every manipulation should be done gently.

### III.—OPERATIONS ON THE NASO-PHARYNX.

In the after-treatment of operations for adenoids it is an undoubted fact that much harm may be done by injudicious interference. Hæmorrhage is free, but soon ceases. No attempt should be made to stop the bleeding by syringing the nose with ice-water, &c., as has at times been recommended. The application of ice or cold water to the forehead is, however, a valuable agent in stopping hæmorrhage from the naso-pharynx, and may be used with impunity. After an operation for adenoids the patient should be placed in bed on his back or side and allowed to sleep off the effects of the anæsthetic. Frequently vomiting of blood occurs at intervals for from six to twelve hours; this is due merely to the blood which has been swallowed, and need cause no alarm.

The naso-pharynx takes on the average ten days to heal completely, and during this time the patient requires a certain amount of care. For the first one or two days it is best to keep him in bed, and to confine him to the house for the next four days. If the weather be fine he may be allowed out during the daytime for the remaining part of the ten days. During the confinement to the house it is of importance that the temperature of the room should be kept equable, and that the patient should be guarded against draughts. Provided hands, sponges, and instruments are aseptic, and the naso-pharynx be left to itself after the operation, there is no reason to expect any case of post-nasal growths to do other than heal quickly and without complication. The meddlesome practice of douching or syringing the nose or naso-pharynx with the idea of keeping the parts aseptic is not only useless but distinctly harmful. Cases in which inflammation and suppuration of the middle ear has occurred after operations for adenoids can, unless due to laceration of the Eustachian cushions, be always traced to either douching or syringing, carelessness in getting into draughts, or unsanitary surroundings.

As regards the feeding of the patient, for the first day he should take nothing but liquid—milk, beef tea, &c., and for the first six hours or so after the operation (whilst there is any vomiting of blood) it is better to only give occasional teaspoonsful of hot water or ice to suck. The second two days soft foods (bread and milk, milk puddings, custards, &c.) may be given, after which the soreness will have passed off sufficiently to allow of his taking more solid food.

Whilst in attendance upon these cases, the nurse will be of considerable help in initiating that after-teaching which is so necessary in cases of adenoids. The mouth-breathing induced by the growths rapidly becomes a habit, and this habit remains unless combated. After the first few days are over, therefore, the nurse should frequently remind the patient to keep his mouth shut, and endeavour to breathe properly through the nose.

Any ear complications (discharges and the like) will be treated during the convalescence as directed by the surgeon. In cases in which politzerisation is needed, however, this must not be done till at least a week after the operation. These remarks may also be taken as applicable to the after-treatment of operations for the removal of other growths from the naso-pharynx, but, at the risk of too frequent repetition, I would again impress upon the reader the evils of syringing or douching the nose after operations in this region; not only does it interfere with the proper rest necessary for repair, but it is a possible source of infection, and may also set up severe inflammation of the middle ear.

### Training in the Provinces.

(*Continued from page* 78.)

### RADCLIFFE INFIRMARY, OXFORD (139 Beds).

Terms of Training.

A course of two years' training is offered to nurses at the Radcliffe Infirmary, Oxford, applicants being required to enter upon a two months' trial before they are finally accepted as probationers. The age considered desirable is from 22 to 30. A premium of £25 is expected from all probationers, and for the first year they receive no salary; for the second year they are paid £10. If not appointed after the trial two months, the premium is returned to the candidate, 10s. 6d. a week being deducted to cover expenses. Probationers retained on the staff at the end of the two years receive a salary of £16 for the third year, and £20 for the fourth year. Sisters' salaries vary from £28 to £32 per annum. Lectures are given to the nurses by members of the medical staff, and certificates are granted on completing the two years' engagement. Laundry, and a certain amount of indoor uniform are provided by the hospital for the nursing staff. Promotion depends upon personal fitness. The nurses take three months' day and three months' night duty alternately.

A limited number of probationers are received for one year's training on payment of a premium of £40, to be paid in advance.

Hours On and Off Duty.

The average hours on duty for staff nurses and probationers amount to about 10 a day, sisters 9½. The day staff go on duty at 7 a.m., and leave their wards at night at 8.30 p.m. Hours off duty are 2 or 2½ hours each day, with time for meals. Sisters, nurses, and probationers alike have one day's leave each month, when they do not go on duty at all. Sisters have a long evening, *i.e.*, 5.30 to 10 p.m., once a week, and a half-day once a fortnight. Nurses have a long evening once a week. Annual holidays are for sisters one month in the year, for nurses three weeks, and for probationers two weeks during their first year, three weeks afterwards. Night nurses are on duty from 9 p.m. to 8.30 a.m., and their recreation time is from 9.30 a.m. to 12 in winter, from 6 30 to 8 30 p.m. in summer.

Meals.

All meals are served in the nurses' dining-hall, and no "allowances" are made except to nurses on night duty. Day nurses' meal-times are: Breakfast, 6.30 a.m. (sisters, 7.45); lunch, 9 to 9 30 a.m.; dinner, 1.30 and 2 p.m.; tea, 5 to 5.30 p.m.; and supper—for nurses, 8.45 p.m.; for sisters, 8 p.m. Night nurses breakfast at 8.40 p.m., and dine at 8.45 a.m.

### PERTH COUNTY ROYAL INFIRMARY (110 beds).

Terms of Training.

Candidates for training at the County and City of Perth Royal Infirmary, to give it its full title, must be between 25 and 35 years of age. The course of training is for two or three years, a certificate being granted after passing examination and satisfactorily completing the engagement with the hospital. Probationers' salaries begin at £12 the first year, rising to £18 the second year, and during the third to £22 or £25, according to the work required of them. Staff nurses, of whom there are four, receive from £25 to £40 per annum, according to length of service and nature of appointment. Laundry and indoor uniform are provided by the infirmary. There are no special or unpaid probationers. Staff nurses are chosen from amongst the nurses trained at the infirmary, the qualifications being at least two years' training and general suitability. Lectures are given by members of the staff to the nurses, during their training, upon elementary anatomy and physiology, and medical, surgical, and fever nursing. The infirmary being, besides a general hospital, fever hospital for the city and county, nurses have all an equal opportunity of gaining experience in infectious work. Night duty is taken by probationers in their second and third years, for periods not exceeding three months at a time, from five to seven being usually on duty at a time, working under a night superintendent.

Hours On and Off Duty.

Probationers go on duty at 7 a.m., and leave their wards at 8.30 p.m.; staff nurses begin their working day an hour later, and remain on duty till 9 p.m. The same hours for recreation are taken alike by probationers and staff nurses, from 1 to 4 p.m. every alternate day, and half a day from 1 p.m. once a fortnight. When the work is light and there is a nurse to spare, they have a whole day or possibly two days all round, as opportunity arises, but this leave is extra. Probationers have three weeks' annual holiday; staff nurses one month.

Meals.

The day nurses begin their day with tea and bread and butter at 6 45 a.m., and the breakfast proper, in which they are joined by the night nurses, is served at 9 a.m. Dinner is at 1 p.m., tea at 5, and supper at 8.30. The night nurses dine at 9.40 p.m. All meals, without exception, are served in the nurses' dining-room, even the night nurses' midnight tea meal, for which they are relieved by the night superintendent. No "rations" are served out to the nursing staff.

### Lectures on Medical Relief.

Under the Auspices of the Charity Organisation Society.

### THE LUNATIC ASYLUM.

On Friday last Dr. Rayner, of Aberdeen, late Superintendent of the Male Department, Hanwell Lunatic Asylum, &c., lectured on "The Lunatic Asylum." Tracing the history of such institutions back to early ages, Dr. Rayner said that the first systematic treatment of the insane was recorded as practised in Mahommedan countries in the twelfth century. In Christian countries insanity was treated as a crime on the ground that the insane person was possessed by evil spirits, and until the early part of the present century lunatics were treated with barbaric cruelty. He then described the processes by which gradually a better system was adopted, the insane safeguarded while placed under control, and proper precautions taken to prevent a too hasty deprivation of liberty, and to secure more humane treatment. Then followed the institution of authoritative supervision by medical inspectors, and inspection by Commissioners in Lunacy, though the number of the former was still inadequate, and remained the same with 102,000 patients as when these did not exceed 32,000. Dr. Rayner then gave minute particulars of the proceedings to be taken in order to place insane persons under control, and of the system which governed the treatment of lunatics. Then came the question, how should improvement in the present system of caring for lunatics and idiots be effected? In the first place, instead of the existing plan of building huge asylums, in which it was impossible to give individual attention to each case (a point on which the lecturer laid great stress), in all cases where the lunacy was not of a dangerous nature the boarding-out system should be tried, and adopted if found to work well. In Scotland the experiment had been tried and found successful. Subsequently it should be extended so as to form colonies, on the lines already adopted with epileptics, and the lunatics lodged in cottage homes or blocks of buildings in different districts. Land should be acquired and laid out, and employment of every variety provided for all patients who were at all capable of work, for idleness was a provocative of the disease. Such a system would, no doubt, require more supervision, and consequent expense, but the extra cost would be to a great extent met by the self supporting nature of the colonies, and it was from the more complete individual supervision that so much might be hoped and expected. Next, it was of the greatest importance that convalescent colonies should also be provided, to which those discharged from the asylums could be sent, instead of being drafted back at once to their own homes. Of the 6,000 lunatics annually discharged from asylums 16 per cent. were lapsed cases, 16 per cent. found their way back to the asylum, no doubt in consequence of the lack of proper supervision and proper employment at home. If there were more individual care there would be far greater hope in a much larger percentage of cases than at present, and there was also no reason to doubt that in many instances lunacy might be prevented altogether if the disease were taken in hand in time in its early stages, while lapses after discharge might often be avoided if there were proper "after care" of the patients.

### Wants and Workers.

Surgical Aid Society.—Nurse Edith, 42, Petherton Road, Highbury, N., will be glad to share letters for the above for the benefit of a boy who requires an artificial foot. The case is a most deserving one.

A Correspondent offers to send The Hospital, weekly, to any nurse unable to afford the yearly subscription. Applications marked "Hospital" in left hand corner of the envelope will be forwarded.

Miss Flint, Bramber Villa, Cavendish Road, Merton, would be glad to forward, carriage paid, several years unbound numbers of "Misericordia" to any nurse who would care for them.

### Appointments.

Leavesden Asylum, Watford, Herts.—Miss E. M. Howell, late head nurse of the Cheshire County Asylum, Macclesfield, has been appointed Matron of the above.

The Infirmary, Havil Street, Camberwell, S.E.—Miss Bessie Cottrill has been appointed Superintendent of Nurses at the above institution. She received her training at the General Hospital, Birmingham, and she has also been a charge nurse and night sister at the Brook Hospital, Shooter's Hill, S.E.

Workhouse Infirmary, Stapleton, Bristol.—Miss L. Gummer has been appointed Superintendent of Nurses at the above infirmary. She was trained, and earned a three years' certificate, at the Liverpool Royal Infirmary, becoming afterwards sister of the women's wards in the Carnarvon and Anglesea Infirmary, Bangor. She has been during the past year at the General Hospital, Bristol, where, after gaining her midwifery certificate, she was engaged on the private staff.

### Minor Appointments.

St. Helens Sanatorium, Lancashire.—Miss M. Smith, who was trained at the Liverpool Royal Infirmary, has been appointed Sister here.

Wilts County Asylum, Devizes.—Miss Blanche McLean, of the Cheshire County Asylum, Macclesfield, has been appointed Assistant Head Nurse of the above.

Royal Infirmary, Bristol.—Miss Rosa Venn, who was trained in the Liverpool Royal Infirmary, has been appointed Sister of the women's surgical wards at the above infirmary.

The Cottage Hospital, Ashby-de-la-Zouch.—Miss Evelyn Mansel has been appointed Matron here. She was trained at Charing Cross and at Guy's Hospital, and was at one time matron of the Cottage Hospital, Easingwold, Yorkshire. She has recently been sister at the Woman's Hospital, Soho Square, W.

The Convalescent Home, Nottingham.—Miss M. I. Graham, who was trained at the Royal Infirmary, Halifax, has been appointed Charge Nurse of this home. Miss Graham has been successively charge nurse of the South-Western Hospital, London; sister-in-charge, Union Infirmary, Bradford; sister in charge, London Fever Hospital; and temporary night superintendent of the Infirmary, Leicester.

### Where to Go.

Colonial Nursing Association.—On Thursday, December 1st, at three p.m., the half-yearly meeting of this association will be held in the Imperial Institute. Lord Loch will preside, and H.R.H. Princess Henry of Battenberg has announced her intention of being present.

Metropolitan Hospital.—On Thursday, December 8th, 1898, at the Town Hall, Shoreditch, a concert will be given by the Stoke Newington Choral Association on behalf of the funds of the above hospital. Nearly 200 performers will take part in it. Tickets from the Secretary, the Metropolitan Hospital, Kingsland Road, N.E.

The National Orthopædic Hospital, Great Portland Street, W.—On December 2nd and 3rd the annual sale in aid of the additional comforts fund of this hospital will be held under the patronage of the Duchess of Marlborough. It will be open from two to six o'clock each day, and the admission fee is 1s. Contributions of articles for sale will be gladly acknowledged by the matron. Mrs. Archibald Little will lecture on the 2nd, at three p.m., on "Foot Binding in China," and at half-past four on "The Position of Women in China." On the 3rd she will give the same lectures, at the same hours, reversing the subjects.

### Presentations.

Miss Vaux, who during the last ten years has held the appointment of Matron to the Birmingham and Midland Eye Hospital, left that institution on the 18th inst. to open a Medical and Surgical Home at Ipswich. She was the recipient of some handsome presents from friends and officials of the hospital, together with many expressions of regret at her leaving.

### Everybody's Opinion.

[Correspondence on all subjects is invited, but we cannot in any way be responsible for the opinions expressed by our correspondents. No communication can be entertained if the name and address of the correspondent is not given, as a guarantee of good faith but not necessarily for publication, or unless one side of the paper only is written on.]

### WANTED, A COPY OF "THE HOSPITAL."

The Matron of the London Throat Hospital writes : In reply to the request from Canada for copies of The Hospital, I shall be very happy to send mine if you have not already received notice to do so from some other subscriber. I can quite understand anyone liking to have The Hospital.

Nurse Mugridge, of the Southend Nursing Institution, writes : In this week's issue of The Hospital I see (in the "Wants and Workers ") a lady in Canada asking for an occasional copy. I am sending one this week, and shall have very much pleasure in continuing to do so. If you will kindly mention this in your next issue I shall be pleased. Whilst writing I should like to add how we all enjoy reading your paper. We find it so instructive and interesting. I have also to thank you for so promptly forwarding the copy every week.

### DOCTORS AND MIDWIVES.

"Tory" writes : Will you kindly read the enclosed letters and give me your opinion in the "Nursing Mirror." If no "self-respecting doctor would make arrangements with a midwife" to attend her abnormal cases how about (for example) the case that was mentioned in the "Nursing Mirror" a few weeks ago about Nurse Heatley, of Fulham, and her practice ? I want to get the real facts, as it seems a complicated affair and doctors do not agree about it. I think part of the secretary's letter is superfluous. I am quite aware what cases a midwife may attend.

Copy Letter from "Tory" to Secretary L.O.S.

"Dear Sir,—I am trying to work up a midwifery practice in Sutton. I, of course, was anxious to make arrangements with two or three of the doctors to attend my cases when necessary. I applied to one doctor (among others) asking him what his terms would be ? If he would wish to take my whole fee, or what arrangement he would make ? His reply is that he cannot undertake anything of the kind, as he 'would be liable to be struck off the rolls for illegal covering.' He also tells me that as it would be illegal for him to enter into any arrangement with an unqualified medical assistant, therefore it would be illegal if he did so with a midwife holding the L.O.S. certificate, that certificate being no legal qualification as the law is at present. Will you be so kind as to inform me if this be so, and if so what the midwives are expected to do with regard to calling in doctors ? Will you also kindly tell me what effect the State Registration of Midwives will have upon us who have already the L.O.S. certificate ? An early answer will oblige."

Copy Answer from Secretary L.O.S.

"Dear Madam,—The Obstetrical Society in giving a certificate to midwives who pass the examination does not countenance the line of practice which you propose. First, the title of L.O.S. which some midwives have assumed is objectionable. The certificate is not a legal qualification. It is only evidence that the holder is qualified to attend cases of normal labour, as recently defined in our 'Rules and Regulations,' of which a copy was forwarded to you. The midwife is expected to fill the place of the less competent so-called midwife who preceded her. She may act as a nurse under a medical practitioner, or she may act alone as midwife under certain hospital and Poor Law appointments, or alone in attendance on soldiers' wives in military stations, or alone in attendance on women who cannot afford to pay a doctor. In any of these cases she is bound to send for a doctor; in all cases as stated in our 'Rules and Regulations,' of abnormal labour, when the relations of the patient would pay the fee of the medical man, but no self-respecting doctor would do it by previous arrangement with a midwife. When State Registration is accomplished you will be qualified to go on the register.—Yours faithfully, A. Hannan, Secretary."

### GYNÆCOLOGICAL NURSING.

"E. M. E." writes : Will you kindly allow me to insert the following in your widely-read nursing journal ? As a late worker at the Chelsea Hospital for Women, I am sorry any patient (if so be she proves to be a *bonâ fide* "sufferer") should so speak of its comforts. I worked as night sister at the Chelsea Hospital for Women for fourteen months, and am, therefore, in a position to be able to state authoritatively that there was never an insufficiency of bed-pans or slippers on any of the floors. As a class, I have always found nurses make the worst patients. Possibly this poor, "ill used" "Sufferer" may be one of the many who have come to the Chelsea Hospital for Women for treatment. Should I ever need such an operation as is performed at this hospital, I should not hesitate to trust myself to the Albany floor.

"One Who Knows" writes : As one well acquainted with the routine of hospitals, I should like to ask "Sufferer" whether on leaving the Chelsea Hospital for Women she made any complaint of the discomfort entailed on her by the alleged insufficiency of bed-pans on the Albany floor. Everyone who knows this hospital is well aware that one of its strictest rules is, that each patient before leaving should be taken by a nurse to the secretary's office, and left there. She is then asked by the secretary if she has any complaint to bring forward, and is invited by him to make any remark she pleases about the hospital in a book provided for the purpose. If "Sufferer" objects that the presence of the secretary in his office had such a terrorising effect upon her that she dared not make a complaint, then surely her obvious remedy was to communicate in writing to the weekly board, a body that never turns a deaf ear to any complaint. It seems strange that she should wait till the publication of Dr. Giles' lectures to formulate her grievance. I fancy most of your readers, like myself, will be disposed to think that the facts on inquiry will not be as she has stated.

"Another Sufferer" writes : I, also, have read with great interest and gratitude the recent lectures on the above subject by Dr. A. Giles, and can testify to the truth of the sufferer's letter of November 19th, viz., the horrible amount of needless pain caused by the long wait for bed-pans, be they slipper or any other shape, if wanted out of regulation time, which is after each meal. As everyone cannot oblige the nurses by using them when they wish, it often gives rise to great inconvenience on both sides, for if a patient is unfortunate enough, through the almost daily administration of white mixture or other purgatives, to require a pan after the luncheon one has gone round, she is herself loth to ask for it, as the doctor is on his round, and she would scarcely wish him to find her thus when he comes to dress her. If she is a novice to ward routine she does ask for it, and is often told : "You cannot have it now, as the doctor is expected." Now the chief fault lies in this, there are not sufficient bed-pans to go round, so one often hears the nurse-probationer's answer, "You really cannot have it now, they are all in use," and so by the time the poor patient gets it it is useless to her till some one of those unfortunate odd times. Then if one is asked for after the dinner round has passed (especially on the top floor), it is either refused point blank, or given in terror that one of the surgeons will pop in and behold the general confusion. Oh, that top floor after dinner ; no rest, no sleep. Again, the matron writing does not evidently know that there are some who make it their rule that no bed-pan be given at meal times. Very right, too ; but there must sometimes be an exception. While a patient I asked for a slipper bed-pan one morning before breakfast, and the night nurse said to me : "It is twenty to six now, and I am getting breakfast, so you can't have it." Well, I had taken two pills the night before, and was only a five days' old operation case, so I waited on as long as I could, and at last rang my bell and begged for that pan. I was then in torture, and I was answered : "It is a most strict rule of the sister's that no bed pan be given at meal times." So I had to endure my torture till the others had breakfasted (I had none, as I could not eat any, so had to wait for food till lunch was served). Now, the question is this : Who made that rule, or was it conjured up by the nurse's powers of fabrication, and why did she not give it at twenty to six a.m.? I found afterwards other night nurses kindness itself, and I thanked God when I went to another floor. Therefore, Dr. Giles can now quote from three patients; indeed, he might truthfully do so from a far greater number of witnesses. If it is not a rule, why is the daily practice of giving bed-pans immediately after food so rigorously and unfailingly carried out? If Dr. Giles was lecturing students he might keep himself exclusively to anatomy and physiology subjects, but when he is kindly teaching probationers the A B C of such subjects surely he may be permitted to tell those young girls who are one day to be nurses what is their duty, especially as they will nurse some of his own cases, and he, having heard complaints from his patients, would be a little less than a man were he to take no notice and let them suffer still from a fault that could and should be done away with. It is his duty to do it, as evidently those who ought to have done so and have either not done it or have proved themselves utterly inefficient and failed ignominiously. The writer thinks, too, that the person who writes (as matron) has well succeeded in teaching insubordination to the authority of those who are her superiors and in whose hands, under God's, are the issues of life and death. The nurses at Chelsea are, as a body, like any other body, mixed, and it requires a wonderful power of discernment in a staff nurse to guide each particular character in the right way. Those under their supervision are like all young people of the day, full of importance, and when the technicalities are learned they think their lesson is learnt. Alas! many have to acquire what Nature has denied them—a soft voice, a gentle hand, and respect for Christ's members, tiresome and unlike their Master though they be. God pity the patients! Some have been nurtured as gentlewomen and seen better days, so that the humiliation of being patients in a general ward adds greatly to their suffering, while the snubs of young girls fall keenly upon them. The chief thing of which to complain is, to my mind, that when a patient has been up for a few hours for, say, two days, she is obliged to tramp up and down a long corridor to a w.c. when almost too weak to crawl, and so exhaust the little bit of strength left in her. The writer cannot speak too highly of the kind and gentle manner of the house physician, the matron, who is ever what a gentlewoman and a true nurse ought to be, and some of the staff; but one or two of the white belts with stringless caps would do well to try and copy them and cultivate their manners, and not try to be superior to their superiors, and so above giving a slipper when a poor probationer is absent.

Dr. Arthur Giles writes: "Matron" evidently feels aggrieved that a medical man should lecture on nursing. I may relieve her mind by telling her that so far from "encroaching on the province of the trained matron," it was at the request of a "trained matron," and, I may add, an exceptionally able one, that the lectures were delivered. Lectures on nursing would appear, moreover, not to be superfluous, since "Matron" herself requires to be taught that though a large slipper is a bed-pan a bed-pan is not necessarily a slipper. If "Matron's" indignation had not interfered with her accuracy she would, perhaps, have quoted me correctly. I did not say, "It is the rule in hospitals," &c., but "It is the rule in some places." "Sufferer's" letter shows that my remarks were not made groundlessly, but to distort what I said into an accusation against nurses of selfishness is mere invention. I venture to think that not one of the many nurses who have worked with me would impute to me such an unkind and unjust charge. My directions as to the taking of fluids by the patient after operation were absolutely plain. Briefly, *if possible* (in the omission of these words "Matron's" accuracy again deserts her), the patient should be induced to take nothing by the mouth for at least twelve hours, but if much distressed by thirst she may be allowed sips of hot water. Where is the contradiction? The instances I related of patients drinking the contents of their hot-water bottles were given to emphasise the need of vigilance in the early days after operation, and I think no one besides "Matron" would construe what I said into meaning that irrational cravings should be gratified. May I commend to "Matron" the excellent plan of reading correctly and with comprehension before criticising. "Sufferer" has referred to the drawbacks she experienced after operation, but takes occasion to exonerate the nursing staff from blame, and to acknowledge their kindness and attention. She mentions the staff of the Chelsea Hospital for Women, and I can cordially confirm all that she says in their praise. I am glad to be able, also, to assure her that the inconveniences to which she refers were but temporary, and have been remedied.

### HOSPITAL HOUSEKEEPING.

"A Lady Superintendent" writes: One would suppose those responsible are unaware that there is other fish than cod in the sea and quite as cheap. In the institution in which I work a contract is made for supply of fish at so much per pound. No kind is specified, yet cod is always sent, and I concluded it was cheaper than other kinds. Would the writer kindly state what other fish would be as cheap, so that we may ask for it, and thus benefit by others' experience.

*** In answer to "Lady Superintendent" on the question raised in some recent articles in The Hospital on "Hospital Housekeeping" and the monotony of the fish and general dishes served to the patients of our institutions "A Late Matron" writes: I am very glad to see that interest is being aroused by my articles on "Hospital Housekeeping," for there is no department more important to the sick than the kitchen and culinary departments. Yet very little interest appears to be taken by the average matron, sister, and nurse in the great food question. As I stated in my articles cod is a by no means economical fish, neither is it nearly so nutritious as many other varieties. As to its palatability tastes differ. At private tables cooked to perfection with a piquant source it is sometimes extremely good. As served in the average hospital it tends to be tough and stringy. No sauce accompanies it, and one quite sympathises with those patients who say, "Now, if I could only have a little vinegar I could enjoy my fish." But vinegar can have no place in a sick dietary, so the uninviting cod is swallowed somehow. Now hake and halibut are decidedly more palatable than cod—both are less expensive and I believe more nutritious—but as I write I have no table of the comparative nutritive values of fish before me, so that I speak from memory. There is a long-standing tradition that the sick person must eat boiled fish; but this, like many popular beliefs, is an entire fallacy. Anybody who can digest boiled cod could just as easily digest baked halibut, or hake, or fried plaice. Only it must be remembered that the outer skin should be removed from fried fish after it is cooked, and, of course, baked fish should also be skinned. It is then perfectly suited to the invalid. Of course, if fish is put into the frying-pan with warm fat it will be soddened and greasy. But the hospital housekeeper must realise that the fish should never be put into the pan till the frying butter or clarified dripping is boiling. If this be attended to and the fish skinned when cooked it will be perfectly suited to a sick person capable of digesting cod. As a practical domestic woman, I stand aghast at the method of cooking the eternal cod as practised in the average hospital kitchen. It is boiled in oceans of water, so that the small amount of nourishment it originally contained is speedily absorbed by the deluge of water in which it floats, and this mode of cooking renders the fish stringy and tough. If it were steamed—a process which is just as easy as boiling—and the small amount of fish liquor which results were added to milk and the whole thickened with arrowroot to form a nice white sauce, even the hospital cod would prove appetising. Even so it is bad nursing and bad housekeeping to give any sick person "on fish" the same fish two consecutive days. Steamed fresh haddock makes an admirable dish with a nice white sauce—and by this I do not mean the lumpy melted butter so dear to the heart of the British cook. Fresh haddock may be bought at the average fishmonger's at 4d. a lb. At contract hospital prices it is so cheap that it could be served all round advantageously to the patients and economically to the hospitals. I am dealing further with this most important question of institutional housekeeping, and I ask on behalf of the sick patients of our hospitals that my sister matrons will take an interest in this, one of the most important departments of nursing, and give any hints, suggestions, or results of their experience to the readers of The Hospital. No patient can be said to be well nursed who is not fed according to the highest dietetic principles.

### For Reading to the Sick.

### THE SAVIOUR'S POWER.

**Verses.**

ARE we not Princes? we who stand
As heirs beside the Throne;
We, who can call the Promised Land
Our Heritage, our own;
And answer to no less command
Than God's, and His alone?

O, God! that we can dare to fail,
And dare to say we must!
O God! that we can ever trail
Such banners in the dust,
Can let such starry honours pale,
And such a blazon rust!

Shall we upon such titles bring
The taint of sin and shame?
Shall we, the children of the King,
Who hold so grand a claim,
Tarnish by any meaner thing
The glory of our name? —*A. Procter.*

My heart was restless, weary, sad, and sore,
And longed and listened for some heaven-sent token;
And like a child, that knows not why it cried,
'Mid God's full promises it moaned, "Unsatisfied!"

Yet there it stands! O love, surpassing thought,
So bright, so grand, so clear, so true, so glorious;
Love infinite, love tender, love unsought,
Love changeless, love rejoicing, love victorious!
And this great love for us in boundless store;
God's everlasting love! What would we more?
*F. R. Havergal.*

**Reading.**

"Mighty to save."—Isaiah lxiii. 1.

This sounds like the language of one who goes forth "conquering and to conquer." No wonder the Jews, with their carnal minds, did not recognise in Jesus of Nazareth the conqueror thus foretold, for they did not, and could not, appreciate the mighty power of four things:

1. The Power of Gentleness: It conquers by winning. It speaks words which we carry away, which act like leaven, changing bitterness to sweetness and wrath to love. . . Christ's gentleness changes everything and makes him "mighty to save!"

2. The Power of Patience: This acts like drop by drop falling upon the rock; it gradually wears away all that hindereth. "Behold, I stand at the door and knock," says Christ. He often stands for years! We know that He is there—we hear the knock. "Let Him knock," we say, "I am busy," or "I will open by and by." No reproach follows, no impatient word is heard. Christ stands till hinges have rusted and weeds have grown. And at length we can resist such patience no longer. The heart gives a great throb, the door is thrown open, and we are at the feet of Him who by patience has proved himself "mighty to save."

3. The Power of Self-Sacrifice: Christ gave himself for us. He laid aside His glory. He became a man of sorrows. He endured the contradiction of sinners. He died upon the cross. Thus "lifted up," He draws all men unto himself and becomes "the power of God unto salvation." His sin-bearing and blood-shedding makes Him "mighty to save."

4. The Power of Example: When our Lord would enrol disciples and enlist followers, his words always were, "Follow me." He left footprints for us—an example that we should tread in his steps. Example gives words their power. It is example in this sense, also, that makes our Lord "mighty to save."—*J. B.*

### Notes and Queries.

**The contents of the Editor's Letter-box have now reached such unwieldy proportions that it has become necessary to establish a hard and fast rule regarding Answers to Correspondents. In future, all questions requiring replies will continue to be answered in this column without any fee. If an answer is required by letter, a fee of half-a-crown must be enclosed with the note containing the enquiry. We are always pleased to help our numerous correspondents to the fullest extent, and we can trust them to sympathise in the overwhelming amount of writing which makes the new rules a necessity.**

**Every communication must be accompanied by the writer's name and address, otherwise it will receive no attention.**

*Previous Trial.*

(95) I have had four months' training in a workhouse infirmary. I tried to the utmost of my power to do my duty there, but was told by the superintendent that I was not fitted to be trained for a nurse, and so had to leave. I have tried several other hospitals, with the result that I cannot gain admission. Can you tell me why it should disqualify me from entering any other infirmary? Is it absolutely necessary for me to say that I have had a previous trial?—*F. S. F.*

Dismissal after a fair trial should suggest that you are really unfitted for the work, in which case failure only can be expected even if you were again admitted as probationer. This does not necessarily reflect discredit on you. Dozens of probationers are rejected for whom the superintendents have a warm admiration, and who succeed admirably in other callings.

*A Young Probationer.*

(96) Will you kindly tell me some children's hospitals in the Midlands where probationers about 18 years of age are taken?—*Amy Taylor.*

You are much too young for any hospital Wait until you are 20.

*Hospital versus Infirmary.*

(97) 1. Can you tell me if a three years' training in a provincial infirmary of 200 beds is equal to that period in a hospital? 2. Whether the Sheffield Royal Infirmary is a recognised training school?—*A. M. M.*

1. "Infirmary" and "hospital" are used indiscriminately in different localities for the same class of institutions, and a three years' training in either is recognised as a sufficient nursing qualification. 2. Have you not confused the Sheffield General Infirmary with the Sheffield Royal Hospital? Both are recognised training schools but as the latter is much the smaller, and only offers a two years' nursing course, the former ranks higher as a training school. Improvements too, are in process which will probably increase the advantages.

*The Right of Refusal.*

(98) Can my committee compel me to take illegitimate maternity cases? They were not mentioned when I was engaged. I have hitherto refused to take them without a doctor being present.

If you are engaged as maternity nurse you must attend all cases which are sent to you by the constituted authority, and you have no right to refuse any.

*Dispensing.*

(99) Kindly inform me as to whether the articles on "Dispensing," published in THE HOSPITAL a short time ago, are now to be had in book form.—*A. C. S.*

"Notes on Pharmacy and Dispensing," by C. J. S. Thompson, 1s., from Scientific Press, London, W.C.

*Training.*

(100) I am 33 years of age, and should like to get into a children's hospital, if I can; if not, into a general hospital. I should want three years' training, with uniform, salary, and certificate. Can you give me the address of some hospitals where they take probationers up to 35 years?—*Probationer.*

You cannot do better than consult "Burdett's Official Nursing Directory," price 5s., from the Scientific Press, 28 & 29, Southampton Street, London, W.C.

*Home for Men in Southern France.*

(101) Information is requested as to any convalescent or nursing homes for young men in the South of France, moderate payment. Particulars and full address of St. John's Home, Mentone; also of the Hollond Institution desired.—*E. B.*

Can any of our readers help "E. B."? We cannot trace address of St. John's Home. The London address of the Hollond Institute is 1, Tavistock Chambers, Bloomsbury, W.C.

*Young Probationer.*

(102) 1. Can you inform me if there is any hospital in London where they receive girls of 18 as probationers? 2. What examinations are they expected to pass on entrance? 3. What length is the term of probation? 4. What salary is given during that time?—*S. (Shepherd's Bush).*

(1) No general hospital will receive so young a probationer. When you are 23, and if you are accepted as probationer, you will be required (2) to pass an examination in elementary subjects, (3) to serve three years (4) at a salary varying from £8 to £14 for the first year to £18 to £22 for the third, according to the regulations of the institution accepting you. Children's Hospitals often accept younger probationers, but the certificate does not cover general nursing.

*Royal National Pension Fund for Nurses.*

(103) Mrs. S. would be obliged if the editor would give her the address of the Royal National Pension Fund for Nurses.

28, Finsbury Pavement, E. [illegible]

*Help for a Governess.*

(104) Where should I write to get help for a governess who, through sickness, has spent all her savings?—*Sister Marrie.*

The Governess Institute, 47, Harley Street, Cavendish Square, W.

